# Adipose Tissue Properties in Tumor-Bearing Breasts

**DOI:** 10.3389/fonc.2020.01506

**Published:** 2020-08-21

**Authors:** Isabelle Miran, Dominique Scherer, Pauline Ostyn, Chafika Mazouni, Françoise Drusch, Marine Bernard, Emilie Louvet, Julien Adam, Marie-Christine Mathieu, Mariam Haffa, Jean-Philippe Antignac, Bruno Le Bizec, Philippe Vielh, Philippe Dessen, Hervé Perdry, Suzette Delaloge, Jean Feunteun

**Affiliations:** ^1^Translational Research Lab, INSERM U981, Université Paris-Saclay, Villejuif, France; ^2^Division of Preventive Oncology, National Center for Tumor Diseases (NCT) and German Cancer Research Center (DKFZ), Heidelberg, Germany; ^3^Institute of Medical Biometry and Informatics, University of Heidelberg, Heidelberg, Germany; ^4^UMR 9019 Genome Integrity and Cancers, Université Paris-Saclay, Villejuif, France; ^5^Breast Cancer Group, Université Paris-Saclay, Villejuif, France; ^6^Biology and Pathology Department, Université Paris-Saclay, Villejuif, France; ^7^Laboratoire d'Etude des Résidus et Contaminants dans les Aliments (LABERCA), UMR 1329 Oniris-INRA, Nantes, France; ^8^Translational Functional Cancer Genomics, National Center for Tumor Diseases (NCT) and German Cancer Research Center (DKFZ), Heidelberg, Germany; ^9^Bioinformatics Core Facility, Université Paris-Saclay, Villejuif, France; ^10^INSERM U669 - Equipe GGS Génomique & Génétique Statistique, Villejuif, France

**Keywords:** breast cancer, adipose tissue, non-cell autonomous, permissive cancer niche, IL-8

## Abstract

The tissue stroma plays a major role in tumors' natural history. Most programs for tumor progression are not activated as cell-autonomous processes but under the conditions of cross-talks between tumor and stroma. Adipose tissue is a major component of breast stroma. This study compares adipose tissues in tumor-bearing breasts to those in tumor-free breasts with the intention of defining a signature that could translate into markers of cancer risk. In tumor-bearing breasts, we sampled adipose tissues adjacent to, or distant from the tumor. Parameters studied included: adipocytes size and density, immune cell infiltration, vascularization, secretome and gene expression. Adipose tissues from tumor-bearing breasts, whether adjacent to or distant from the tumor, do not differ from each other by any of these parameters. By contrast, adipose tissues from tumor-bearing breasts have the capacity to secrete twice as much interleukin 8 (IL-8) than those from tumor-free breasts and differentially express a set of 137 genes of which a significant fraction belongs to inflammation, integrin and wnt signaling pathways. These observations show that adipose tissues from tumor-bearing breasts have a distinct physiological status from those from tumor-free breasts. We propose that this constitutive status contributes as a non-cell autonomous process to determine permissiveness for tumor growth.

## Introduction

Human malignant cells are selected through a multistep process involving successive combinations of cell-autonomous and non-cell-autonomous oncogenic events. Cell autonomous events are of genetic or epigenetic nature. The human cells genome undergoes persistent mutagenic events that are either extrinsic (caused by exposure to exogenous agents) or intrinsic (caused by products of the proper cell metabolism or by defective DNA homeostasis). Recent mathematical modeling has estimated that 70–90% of the causal factors driving the most common cancers are “extrinsic” (1). The integrity of the genome is efficiently maintained by the DNA damage repair machinery (DDR). Among the lesions which escape this surveillance and become acquired mutations, some are drivers which bring about new potentially oncogenic properties, some are passengers and harmless for the cell.

Non-cell autonomous events represent the intervention of the tissue stroma as a physiological tissue niche. The tissue stroma is described as macroenvironment as opposed to microenvironment, which represents the immediate proximity of a hosted tumor. The constitution of macroenvironment is complex and organ-specific. It plays an important role in the normal physiology of the organ but also in the natural history of tumors. First, as a source of potentially mutagenic metabolites, it is believed to participate in the initiation steps of malignant transformation. The adipocyte compartment stores and releases lipophilic substances, including powerful mutagens, such as endogenous metabolites and persistent organic pollutants (POP) (2), thus exposing proximally embedded target tissues to mutagenic events. Second, it allows emerging tumor cells to develop their invasive potential, locally and ultimately in metastatic sites. As pointed out by Dongre and Weinberg (3), important programs for tumor progression such as epithelial mesenchymal transition (EMT) are not activated as cell-autonomous processes but under the conditions of cross-talk between tumor and stroma.

The role of the macroenvironment has fostered the concept of etiologic field effect (4). As an example, the mammary gland is composed of epithelial cells, representing the functional compartment of the organ, embedded in an array of fibroblasts, fat, immune and angiogenic cells each playing a specific role in the organ physiology. Mammary tumors originate from malignant transformation of epithelial cells but their outgrowth and dissemination properties rely strongly on the “permissiveness” of the macroenvironment (5). A direct illustration of the cancer field concept has been provided by the elegant work of Sflomos et al. (6) which pointed to the epithelial environment as a determinant of luminal phenotype and hormone response in human mammary tumor cells transplanted into the mouse mammary gland. Similarly, the importance of stroma properties in initiating mammary tumor development is emphasized by the observation that tumors grow in cleared rat mammary fat pads treated with carcinogen, regardless of whether the injected epithelial cells were treated with carcinogen *in vitro* (7). Finally, the demonstration of cancer-driving mutations in phenotypically normal tissues strongly supports the contribution of non-cell autonomous oncogenic events (8).

Just alike mutations in DDR genes, which are associated with hereditary predispositions to various cancers, the polymorphism of stroma components, may as well define the constitutive permissiveness (predisposition) of tissues for the development of cancer (4). Indeed, the concept of mesenchymal niche-driven oncogenesis has been largely documented in the hematopoietic organs in which multiple cases of association of germline mutations in bone marrow environment with dysregulated hematopoiesis have been reported (9).

Distributed into various compartments of the human body as part of the stroma, peripheral adipose tissues, long believed to be no more than an energy storage organ, are actively involved in cross-talking within tissues with functional cells and other components of the stroma. As such they play a crucial role in the physiology of healthy organs but also in pathological contexts such as diabetes and cancer. A large body of literature has illustrated these cross-talk properties in cancers (10). The enhanced risk of breast cancer in obese women has been long recognized (11). It is best explained by the intervention of obese-specific adipose tissues (12–14). They accumulate and release highly reactive tumor-promoting activities (reactive oxygen species; estrogen metabolites…) capable to initiate malignant transformation of adjacent epithelial cells (15) and key mediators, which eventually bring about permissive conditions for the progression into invasive carcinomas (16). At the invasive front of the tumor, adipocytes and tumor cells are neighbors. These adipocytes, called as cancer-associated adipocytes (CAA), display specific phenotypes (10, 17) including: (i) dedifferentiation, (ii) release of adipocytokines, proinflammatory cytokines, growth stimulating molecules (insulin and/or insulin-like growth factors) (18, 19), and (iii) metabolic remodeling (15, 20). Among adipocytokines, leptin and adiponectin are believed to play a major role in breast cancer onset and/or progression. Rappaport (21) and Rappaport and Smith (22) have proposed that the human exposome should be characterized by measuring important constituents of the body's internal chemical environment arising from both exogenous and endogenous sources. This would represent a powerful basis for evaluating environmental exposures and cancer risk (23).

The analysis of exposomes is particularly relevant to adipose tissues because of the extended half-life (~8 years) of adipocytes (24) and their capacity for storage of lipophilic substances. As such, they witness endogenous and exogenous exposures to various carcinogens, endocrine chemicals, inflammation, and cellular activation processes. Presumably the capacity to store and release these agents is polymorphic and therefore individually determined. Hence, comparing breast adipose tissues from tumor-bearing and tumor-free breasts may uncover parameters that define degrees of constitutive cancer permissiveness.

The present study evaluates the morphological and functional properties of constitutional adipose tissues from tumor-bearing (adjacent or distant from the tumor) and tumor-free breasts. We wish to emphasize that the sampling has excluded the cancer-associated adipocytes (CAAs) present at the tumor front.

The ultimate aim of the present study is to define a signature of cancer permissiveness that may be translated into biomarkers of individual risk of breast cancer or ongoing carcinogenesis. The following parameters of the adipose tissues were evaluated: (i) adipocytes size and density, (ii) status of inflammation and vascularization, (iii) secretome capacity, and (iv) gene expression.

## Materials and Methods

### Patients

Two cohorts of participants from a single center have been prospectively selected according to the criteria summarized in Table 1 and Supplemental Figure S1. All women signed an informed consent form prior to surgery. The study has been approved by the Institutional review board. Women with breast cancer were eligible for the present study if they were planned to undergo mastectomy (partial or total) for the purpose of the removal of a proven unifocal untreated intraductal or invasive carcinoma, if they had no history of other malignancies of the breast, or of other malignancies within the past 5 years. They were required not to have received any therapy prior to surgery. Women with tumor-free breast were eligible if they were planned to undergo breast surgery for a benign lesion or for breast reduction and had no personal history of cancer or atypical lesion. Relevant clinical information (including family history, previous biopsy for begnin lesion, bra cup size, BMI, menopausal status, diabetes, dyslipidemia, hormonal therapies, tumor's characteristics) were prospectively collected for all individuals. Germline *BRCA1/2* mutation carrier women were excluded.

**Table 1:** Patients and samples characteristics.

	**Breast cancer carriers**	**Non cancer women**	***p***
***N***	43	6	
Age (median) (range), mean (sd)	67.5 (40–93); 67 (13.3)	47 (26–51); 43.4 (9.0)	10^−4^
BMI (median) (range), mean (sd)	27.5 (19.1–38.2); 27.8 (5.2)	25.1 (21–29.6); 25.2 (4.5)	0.33
Family history of breast cancer	10/43 (23.2%)	3/6 (50%)	
Age at menarche (med)	13 (10–17)	13 (11–14)	
Menopausal	32/43 (74.4%)	0	
Taking exogenous hormones at diagnosis (pill or HRT)	13/43 (30.2%)	0	
History of previous breast biopsy	12/43 (27.9%)	1	
Diabetes	8/43 (18.6%)	0	
Dyslipidemia	15/43 (34.9%)	0	
Vascular disease	7/43 (16.3%)	0	
Current smoker	5/43 (11.6%)	2	
Type of cancer		–	
DCIS	2,00	–	
Invasive	41,00	–	
ER expression	29/43 (67.4%)	–	
PR expression	27/43 (62.8%)	–	
HER2 overexpression or amplification	2/43 (4.6%)	–	
Node-positivity	13/43 (30.2%)	–	
Type of breast surgery		–	
Lumpectomy	31/43 (72.1%)	5/5 (100%)	
Mastectomy	11/43 (25.6%)	–	

*p = Welsch test*.

### Sample Collection

In the case cohort (tumor-bearing breasts), two subcutaneous adipose tissue samples of ~1–1.5 cm^3^ were harvested for the purpose of the study, respectively at a distance of 0.5–1 cm (adjacent sample: AdipTa) and more than 5 cm (distant sample: AdipTd) from the tumor. For three cases, an additional AdipTa sample was obtained to evaluate peritumoral heterogeneity. In the control cohort (tumor-free breasts), a single “normal” adipose tissue sample of ~1–1.5 cm^3^ (AdipN) was collected as part of the surgical sample.

The samples were immediately processed. They were macroscopically dissected to remove as much normal epithelial and tumor tissues as possible. Samples were cut longitudinally in half. The first half was formalin-fixed and embedded in paraffin (FFPE) and included for histological and morphological analysis and the second half was cut in three equal parts for: (i) gene expression analysis (snap frozen and stored at −80°C), (ii) secretome analysis (dropped in culture medium), (iii) steroidome analysis (snap frozen and stored at −80°C), that has been the matter of a previous dedicated published work (25). HES stained sections of FFPE blocks were examined to evaluate the epithelial cells content. Samples containing more than 5% of epithelial cells were discarded.

### Morphology of Breast Adipocytes

Morphological parameters of adipocytes in tumor-bearing and tumor-free breasts were studied on 3 μm thick FPPE sections and stained with Acustain Elastic stain. The slides were scanned with a 20x magnification on an Olympus VS120. Definiens Developer software (Trimble) was used to create an algorithm allowing the recognition and the morphological study of adipocytes on large tissues. Measures were performed on down sampled images (25%). Images divisions were 4,000 × 4,000 pixels blocks. Block stitching and post-processing allowed evaluation of several parameters on patient's samples: number of adipocytes/area unit, mean adipocytes area. The distribution of adipocyte sizes and density in AdipTa and AdipTd were compared by a paired *t*-test and a Pearson correlation test (t.test and cor.test R functions). Their distribution between AdipN and AdipTa (the sample adjacent to the tumor) were compared using a *t*-test (t.test R function).

### Immunohistochemistry

Paraffin sections of FPPE samples were processed in a fully automated staining instrument ULTRA (Ventana Medical Systems) using the Ultra-view kit. The following antibodies were used: Anti-human CD68 clone KP1 from DAKO (1/1,000) detects a monocyte/macrophage associated antigen. Anti-human CD34 clone QBEnd/10 from Novocastra (1/40) predominantly stains endothelial cell membranes. Anti-human CD163 clone 10D6 from DBS (1/75) is specific to the monocytic-macrophage lineage. Anti-human Mast Cell Tryptase (MCT) clone AA from DAKO (1/1,600) is specific for mast cells. Slides were scored by consensus of four simultaneous experienced readers and graded from −2 to +4 by comparison with adipose tissues from a normal breast reduction reference. Means and standard deviations of the four scores in samples AdipN, AdipTa, and AdipTd were computed. A paired Wilcoxon test was performed to test for differences between AdipTa and AdipTd and a Wilcoxon test to test for difference between AdipN and AdipTa. Spearman's correlations ρ between scores in samples AdipTa and AdipTd were computed, and their significance was tested using the cor.test R function [R Core Team (2018). R: A language and environment for statistical computing. R Foundation for Statistical Computing, Vienna, Austria. URL https://www.R-project.org/].

### Secretome Analysis

The capacity of fat tissue samples to secrete cytokines and other growth factors was evaluated as described in Caer et al. (26). In brief, the samples were weighted and dropped in culture medium (1 mL of medium per 0.1 mg of tissue). The medium was: ECMB medium (Endothelial Cell Basal Medium, Promocell C-22220) supplemented with 1% fatty acid-free serum albumin (PAA K41-002) and 1% penicillin/streptomycin. After 1 h at 37°C in 5% CO_2_ atmosphere, the medium was replaced by fresh medium and incubation pursued for 24 h. The medium was then collected on ice and centrifuged for 10 min at 10,000 rpm at 4°C. Supernatants were stored at −80°C for subsequent analysis.

The cytokines secreted in the culture medium were assayed with an Elisa kit on MAGPIX (Luminex), using Bio-Plex Pro Human Cytokine Standard Group II reagent, Group II, BIO Plex Pro Human diabetes Leptin, Adiponectin BIO-RAD. Cytokines analyzed were IL-6, IL-8, MCP-1, VEGF, leptin, adiponectin, HGF. The distributions of secretion values in AdipTa and AdipTd were compared by a paired *t*-test and a Pearson correlation test (t.test and cor.test R functions) on log2-transformed values. Association analyses between cancer status and log2-transformed secretion values were performed using logistic regression procedures for fat specific cytokines, angiogenesis factors, and inflammatory markers. Additionally, univariate association analyses were performed for all the variables. For tumor bearing samples, the mean of measures in AdipTa and AdipTd was used in all analyses.

### Correlations Between Secretome and Immunohistochemistry

We used linear models to test for correlations of IHC (immunohistochemistry) scores and secretion of inflammatory markers. Specifically, we tested whether the IHC values are correlated with the inflammatory marker values in AdipTa samples (on the log2 scale) for all combination of variables.

### Gene Expression

Analysis was carried out on one set of breast adipose tissues sampled from a single batch of tumor-bearing breasts. They include 14 AdipTa samples close to (≈0.5–1 cm) and 21 AdipTd samples distant from (>5 cm) the tumors. One set of AdipN samples (*n* = 5 samples) were from tumor-free breasts. Gene expression was measured from RNA isolated from fresh frozen adipose tissue using the Illumina HumanHT-12 BeadChip. Quantile normalized expression values were analyzed between (AdipTa) and (AdipTd) as well as between (AdipTa+AdipTd) and (AdipN) using a weighted *t*-test. The HeatMap corresponds to an unsupervised analysis (ward method) on the basis of the 177 probes (representing 137 genes) defined by the differential expression at a parametric *p*-value of 0.001, between tumor–bearing (AdipTa+AdipTd) and normal (AdipN) samples.

The expression data have been submitted to the Array-Express repository (http://www.ebi.ac.uk/arrayexpress) under the accession number: E-MTAB-8638.

## Results

### Participants and Samples Characteristics

Participants' selection flow chart is shown on Supplemental Figure S1 and their characteristics in Table 1. Case cohort included 43 women median aged 67.5 (40–93), median BMI 27.5 (19.1–38.2). The cancer-free cohort included 6 women operated either for benign lesions (*n* = 5) or for mammary reduction (*n* = 1), median age 47 (26–51), median BMI 25.1 (21–29.6). The heterogeneity within the participants is noteworthy. First, heterogeneity within the case cohort across multiple statuses such as age, menopause, or hormone receptors statutes for ER/PR tumors; second differences between case and control cohorts: age (*p* =10^−4^), menopause and other pathological conditions. It is also noteworthy that BMIs are not significantly different between the two cohorts (p = 0.33).

### The Overall Morphology of Adipose Tissues Is Similar in Tumor-Bearing and Tumor-Free Breasts

**Figure 1:**
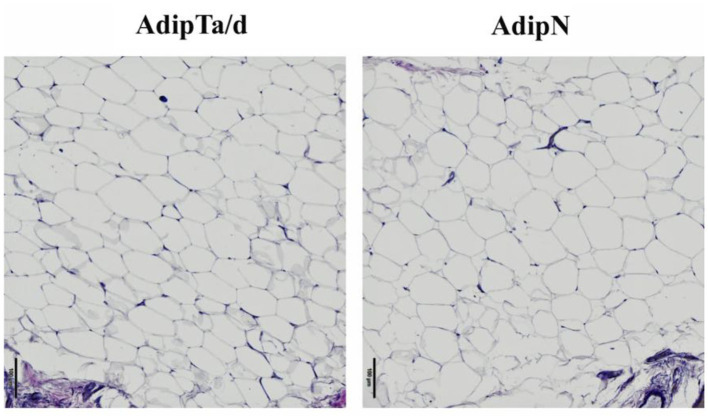
Representative images of adipose tissues from AdipN (tumor-free breasts) and AdipTa or AdipTd (tumor-bearing breasts).

**Figure 2:**
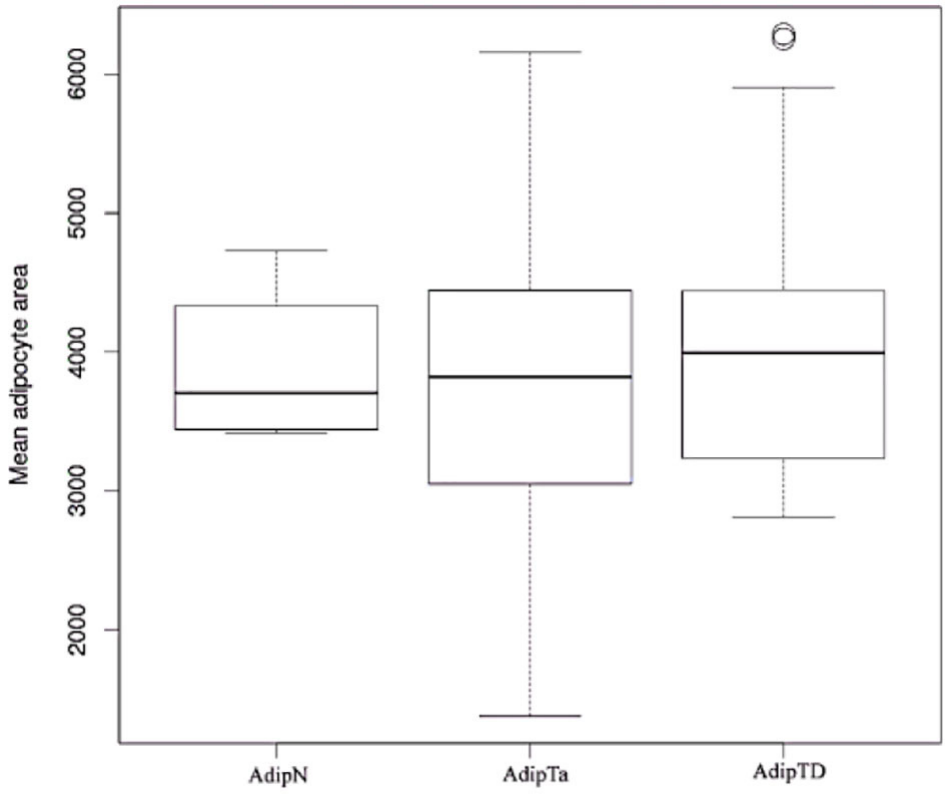
Box plots images: Boxplots comparing sizes of adipocytes in AdipN vs. AdipTa and AdipTd.

The size and density of adipocytes were analyzed in a series of 47 samples including 21 pairs of AdipTa+AdipTd from the same patients, 3 AdipTa alone and 2 AdipN (from tumor-free women). Representative images of AdipN, AdipTa/d adiposes tissues presented on Figure 1 show no difference in morphology, size or density between the three types of sample. This is confirmed by the statistical analysis presented on Figure 2 and Table 2 which show that the distribution of the scores in samples AdipN, AdipTa, and AdipTd are not significantly different. Altogether these results show that from a distance >0.5 cm from the tumor, the adipose tissue is morphologically homogeneous. Furthermore, none of the Adip samples, regardless of the women' BMI, displayed crown-like structures (CLS), alike those which have been described in obese breast and in tumor-bearing breast adipose tissues at the invasive front of the tumor (10, 16, 27, 28) and also in menopaused women (29). The absence of these structures confirms that none of the samples from cancer-bearing breast (AdipTa or AdipTd) are of the obese type, and that the invasive fronts of the tumors have indeed been excluded in the sampling.

**Table 2:** Size characteristics of adipocytes.

	**A**	**B**	**C**	**D**	**E**	**F**
	**Mean (sd) AdipTa**	**Mean (sd) AdipTd**	**Paired *t*-test (AdipTa vs. AdipTd)**	**Pearson r (AdipTa/AdipTd)**	**Mean (sd) AdipN**	***t*-test (AdipN vs. AdipTa)**
Mean area (μm^2^)	3798 (1,115)	4026 (999)	0.2	0.87 (5 10^−9^)	3830 (542)	0.9
Media area (μm^2^)	3141 (1,019)	3366 (878)	0.2	0.84 (4 10^−8^)	3276 (611)	0.7
Density/mm^2^	151 (32)	155 (29)	0.3	0.51 (7 10^−3^)	164 (16)	0.2

*A and B: means and standard deviations of the scores in samples AdipTa and AdipTd. C: p-values of paired t-tests between these two samples. D: Pearson's r (p-values in parenthesis) between AdipTa and AdipTd. E: means and standard deviations for sample AdipN. F: p-values of t-test between AdipN and AdipTa*.

### The Overall Immune Cell Infiltrates and Neovascularization Markers Is Similar in Tumor-Bearing and Tumor-Free Breasts

**Figure 3:**
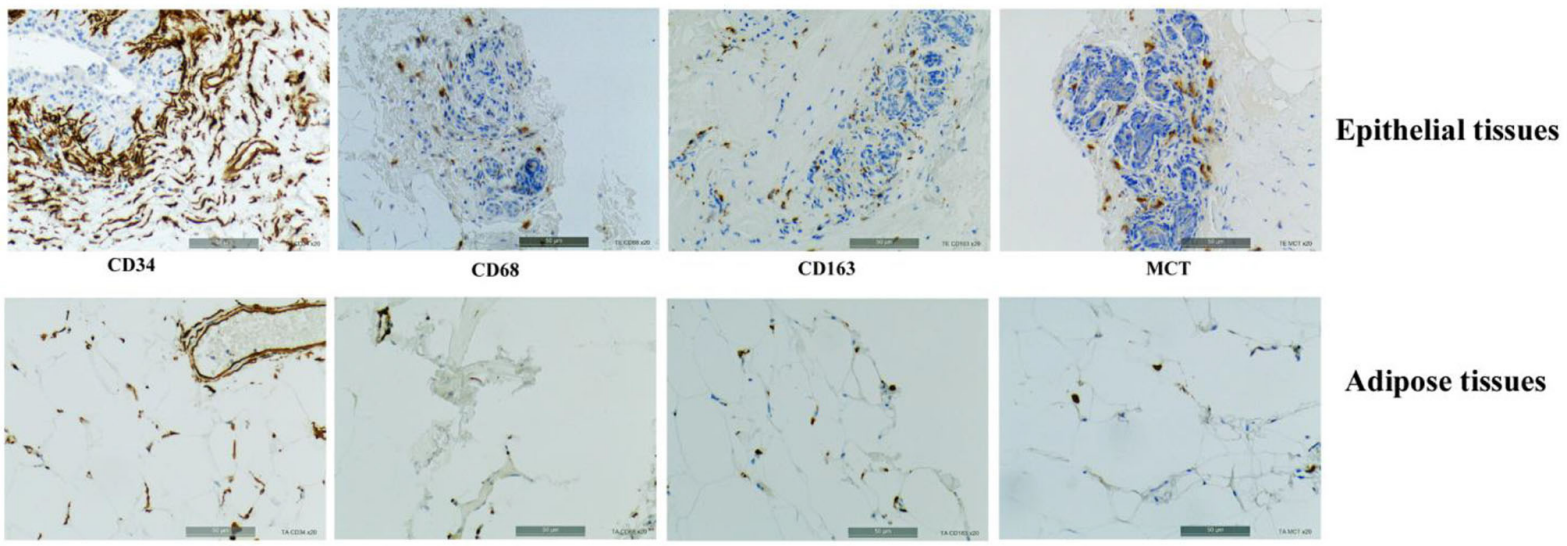
Representatives IHC images of adipose tissues and epithelial tissues (as controls) stained, respectively, with C34 predominantly staining endothelial cell membranes, Anti-human CD68 staining monocyte/macrophage, Anti-human CD163 specific to the monocytic-macrophage lineage, and Anti-human Mast Cell Tryptase (MCT).

**Figure 4:**
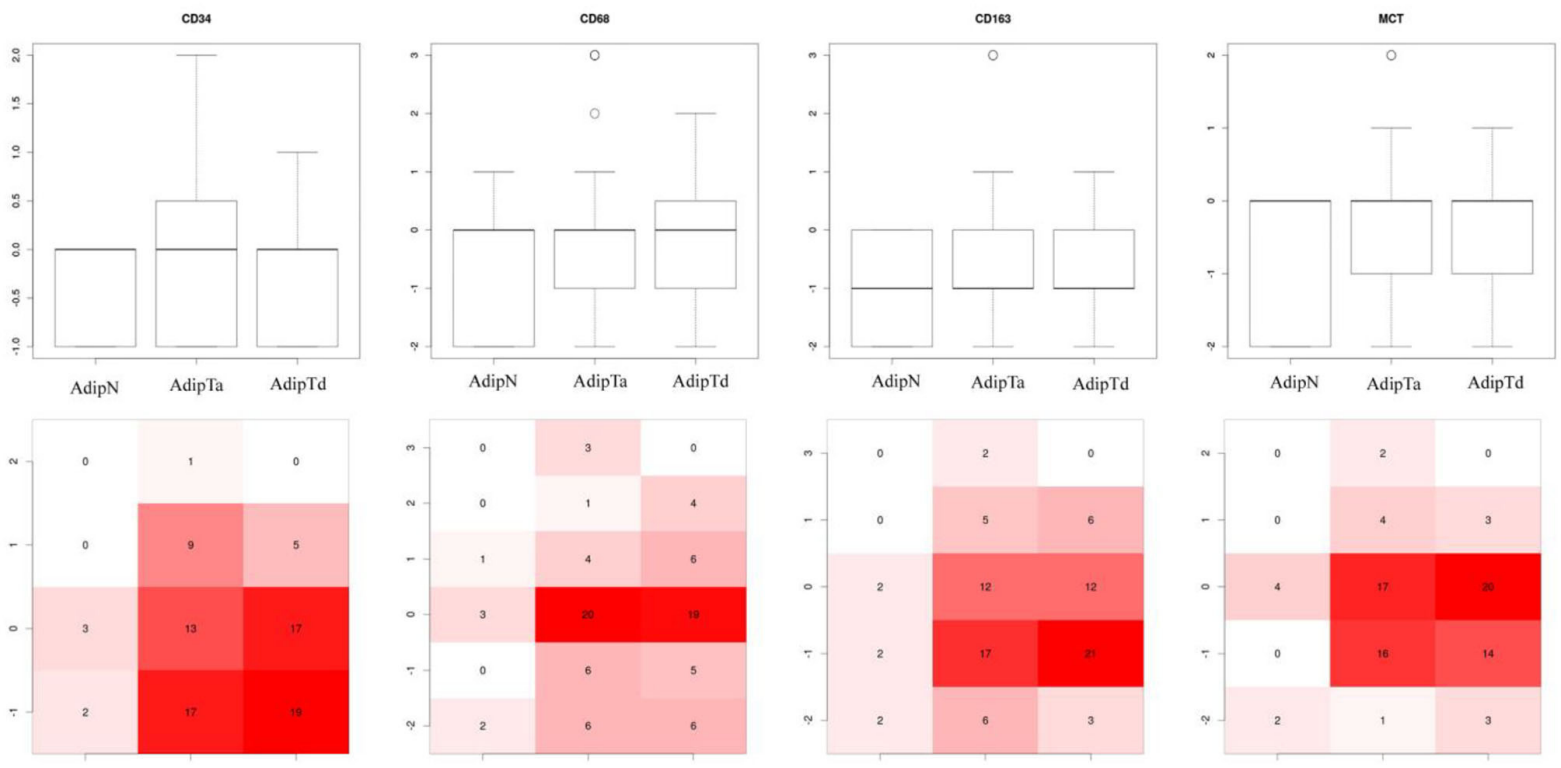
Box plots of distribution of IHC markers in the three sets of samples. AdipN, adipose tissues from tumor-free breasts; AdipTa, adipose tissues adjacent to cancer; AdipTd, adiposes tissue distant from cancer from the same breast as AdipTa. The bottom part of the figure represents the distribution of IHC scores. The intensity of the color (from white, pink to full red) increases with the fraction of samples in each scoring zone.

Potential differences in the physiology of adipose tissues in the two cohorts were explored with a set of IHC markers for immune cell infiltrates [CD68, CD163, and Mast cell tryptase (MCT)], and vascularization (CD34). Figure 3 shows representative IHC images of adipose tissues and epithelial tissue as controls stained for the four markers. The intensities of labeling were scored on a scale from −2 to +4 integral values. Figure 4 presents the box plots of distributions of the 4 markers within the three sets of samples (AdipN, AdipTa, and AdipTd) and Table 3 presents the statistical analysis. A Wilcoxon paired-test reveals no significant difference between the IHC scores at the two sampling locations in the tumor-bearing breast (AdipTa and AdipTd). Furthermore, these scores are well correlated as demonstrated by a Spearman's ρ correlation test. Similarly, the distribution of scores in AdipN and AdipTa/d were not significantly different. No evidence of excess staining by CD68 which would have signified the presence of crown-shaped structures was observed in AdipTa and AdipTd samples. Altogether, these results lead to the conclusion that the presence of the tumor does not affect overall immune cell infiltration and vascularization status of the adipose tissue at a distance larger than 0.5 cm. We wish to emphasize again that the samples were macroscopically dissected to remove as much normal epithelial and tumor tissues as possible thereby discarding cancer-associated adipocytes.

**Table 3:** Immunochemistry scores.

	**A**	**B**	**C**	**D**	**E**	**F**
**IHC marker**	**Mean (sd) AdipTa**	**Mean (sd) AdipTd**	**Wilcoxon paired test (AdipTa vs. AdipTd)**	**Spearman's ρ (AdipTa/AdipTd)**	**Mean (sd) AdipN**	**Wilcoxon test (AdipN vs. AdipTa)**
CD34	−0.15 (0.86)	−0.34 (0.69)	0.2	0.43 (5.10^−3^)	−0.33 (0.51)	0.8
CD68	−0.07 (1.29)	−0.07 (1.14)	0.9	0.62 (2.10^−5^)	−0.57 (1.13)	0.4
CD163	−0.43 (1.17)	−0.50 (0.83)	0.7	0.58 (5.10^−5^)	−1.00 (0.82)	0.2
MCT	−0.25 (0.87)	−0.42 (0.75)	0.1	0.51 (1.10^−3^)	−0.71 (0.95)	0.4

*A: means and standard deviations of the scores in samples AdipTa and AdipTd. C: Wilcoxon paired test (AdipTa vs. AdipTd). D: Spearman's ρ (p-values in parenthesis) between AdipTa and AdipTd. E: means and standard deviations for sample AdipN, and the 6th column the p-values of Wilcoxon test between AdipN and AdipTa. F: p-values of Wilcoxon test between AdipN and AdipTa*.

### Secretion of IL-8 Is Significantly Higher in Cancer-Associated Adipose Tissues Than in Tissues From Tumor-Free Breasts

Fat specific cytokines (Leptin, Adiponectin), angiogenesis factors (VEGF, HGF), and inflammatory factors (MCP1, IL-8, IL6) secreted over 24 h in culture medium were measured by ELISA. All ELISA measurements were log2-transformed. Supplemental Figure S2 and Supplemental Table S1 show that secretomes were highly correlated withS each other in the two locations AdipTa and AdipTd. Thus, at least for these three families of secreted factors, the respective position of the adipose tissue *vis-a-vis* the tumor does not impact on their secretion capacity.

**Figure 5:**
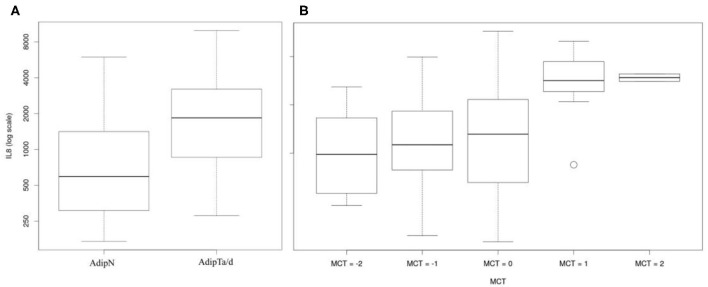
IL-8 secretome analyses: **(A)** Boxplot comparing the secretion capacities of IL-8 by non-tumor-bearing samples (AdipN) and tumor-bearing samples (AdipTa/d). **(B)** Boxplots illustrating the correlations between the capacity to secrete IL-8 and MCT the level estimated by IHC.

Multivariate logistic regressions of secretion levels of fat specific cytokines (Leptin, Adiponectin), angiogenesis factors (VEGF, HGF), and inflammatory markers (MCP1, IL-8, IL6) in AdipTa and AdipTd vs. tumor-free tissue (AdipN) are presented in Table 4. For the majority of the factors tested, no difference was observed in the levels of secretion between fat tissues from tumor-bearing (AdipTa+AdipTd) and tumor-free breasts (AdipN). One exception was the secretion of IL-8, which was significantly higher in cancer-associated adipose tissues than in tissues from tumor-free breast patients (*p* = 0.01) (Table 4C and Figure 5A). Univariate logistic regression analyses confirm that the secretion value of IL-8 is the only one associated with tissue type (*p* = 0.04) (Table 5). The reported odds ratio (OR) for the association between the status of bearing-cancer breast and IL-8 was 1.90, indicating that the odds of a sample being from a cancer case increased by almost 2-fold when secreted concentration of IL-8 is doubled. Although obesity could possibly modify secretion ability, the BMI differences between cases and controls shown to be statistically non-significant is unlikely to account for this result. This result remains significant when the AdipTa+AdipTd cohort is restricted to the 25 patients carrying ER+PR+ tumors instead of the 43 patients in the complete set (*p* = 0.03 instead of *p* = 0.01 with the complete set in multivariate logistic regression analysis). However, the difference between AdipTa+AdipTd and AdipN secretion capacity become non-significant (*p* > 0.05) in univariate regression analysis) (data not shown). This effect is likely due to the major reduction in sample size.

**Table 4:** Multivariate logistic regressions for fat specific secreted cytokines (A: Leptin, Adiponectin), angiogenesis factors (B: VEGF, HGF), and inflammatory markers (C: MCP1, IL-8, IL6) by AdipTa and AdipTd vs. AdipN. Outcome is the cancer status.

**A**	**Regression coefficients (sd)**	***p*-values**
Leptin	−0.47 (0.35)	0.18
Adiponectin	0.03 (0.41)	0.95
**B**	**Regression coefficients (sd)**	***p*****-values**
VEGF	0.44 (0.48)	0.36
HGF	−0.64 (0.62)	0.30
**C**	**Regression coefficients (sd)**	***p*****-values**
MCP1	−0.76 (0.65)	0.24
IL-8	2.16 (0.85)	0.01
IL6	−1.36 (0.83)	0.10

*The reported p-values are Wald test*.

*A: Difference between model deviance and null model deviance is 2.4, leading to a non-significant overall p = 0.29*.

*B: Difference between model deviance and null model deviance is 1.11, leading to a non-significant overall p = 0.56*.

*C: Difference between model deviance and null model deviance is 10.85, leading to a significant overall p = 0.01*.

**Table 5:** Univariate logistic regression analyses.

	**OR (95% CI)**	***p*-value**
Leptin	0.62 (0.28–1.07)	NS
Adiponectin	0.94 (0.45–1.85)	NS
VEGF	1.03 (0.58–1.82)	NS
HGF	0.81 (0.38–1.67)	NS
MCP1	1.15 (0.66–2.05)	NS
IL-8	1.90 (1.03–4.01)	0.04
IL6	1.20 (0.62–2.31)	NS

*Outcome is the cancer status. Secretion values are log2 transformed. NS = p > 0.05*.

### The Mast Cell Tryptase Is a Strong Predictor of IL-8 Secretion Capacity

Association between IHC markers and secretome was estimated using linear models as described in the methods section (Supplemental Table S2 and Figure 5B). The mast cell tryptase score (MCT) is significantly associated with secreted concentrations of MCP1, IL-8, and IL6 and appears as a strong predictor of IL-8 secretion capacity (*p* = 0.006). This association suggests that the secreted IL-8 originates at least partly from the mast cells compartment.

### Gene Expression Signatures in Adipose Tissue From Tumor-Bearing Breasts

Gene expression analysis was carried out on a single batch of 40 breast adipose tissue samples, including 35 samples from tumor-bearing breasts AdipT (14 AdipTa and 21 AdipTd) and 5 samples from tumor-free breasts AdipN.

**Figure 6:**
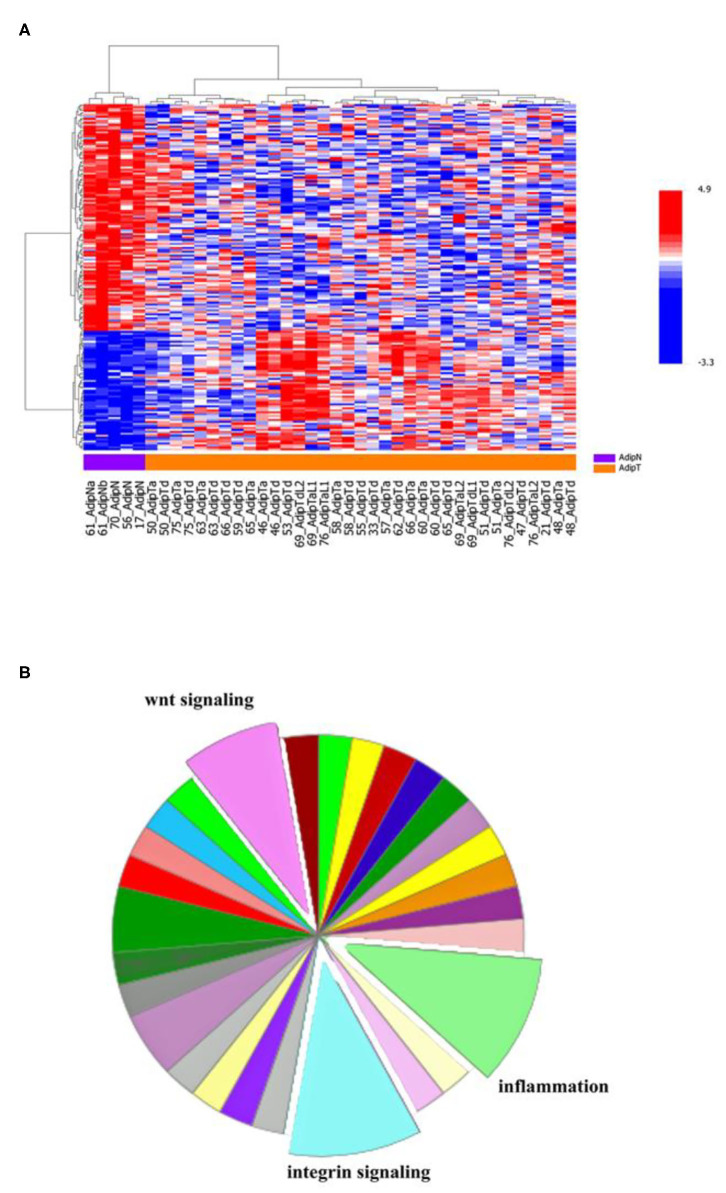
Expression profiles in adipose tissues from tumor-free vs. tumor-bearing breasts. **(A)** HeatMap: Analysis performed on one set of breast adipose tissues sampled from a single batch of tumor-bearing 40 breasts. They include 14 AdipTa and 21 AdipTd samples. One set of AdipN samples (*n* = 5 samples) were from tumor-free breasts. The HeatMap illustrates an unsupervised analysis (ward method) on the basis of the 177 probes defined by the differential expression at a parametric *p*-value of 0.001, between tumor–bearing (AdipTa+AdipTd) and normal (AdipN) samples. **(B)** Panther pathways analysis: the pie chart is the result of an analysis of pathways for the 137 differential genes as referenced in the Materials and Methods section.

The selection of these samples had no biological rationale. They were collected as a first set, passed quality controls (epithelial content, RNA quality) and were analyzed as a single batch. The other samples collected and analyzed at different time points were not included in this analysis to avoid batch effects. The HeatMap shown on Figure 6A represents the expression of 137 genes (177 Illumina probes) which are differentially expressed between AdipTa+AdipTd and AdipN at a threshold of *p* = 0.001. AdipTa and AdipTd clusterize together with some heterogeneity that could not be linked to any of the patient statutes such as age, menopause, or ER/PR. When AdipTa and AdipTb doublets of the same patient are analyzed, the majority (10/13) are side by side, indicating that the expression profiles of adipose tissue adjacent and distant from the tumor are very similar. The five AdipN samples clearly clusterize apart from the set of AdipTa+AdipTd samples. The Panther classification (30), based on the 137 genes differentially expressed between AdipT and AdipN is presented on Figure 6B. It highlights the predominance of three among ~30 general pathways: (1) inflammation mediated by chemokine and cytokine, (2) integrin, and (3) WNT signaling pathways each representing ~10–15% of the genes. The differentially expressed genes are listed in Supplemental Table S3 (Excel sheet). Noteworthy among the genes involved in inflammation pathways are: *CCL8* [chemokine (C-C motif) ligand 8]; *CISH* (cytokine inducible SH2 containing protein); *CD163, CD14*, and *LGMN* (Legumain) which signs the monocytic-macrophage lineage (31)*; MFAP5* (microfibrillar associated protein 5) involved in obesity-associated adipose tissue and extracellular matrix remodeling and inflammation (32); *CILP* (cartilage intermediate layer protein-1) involved in articular cartilage inflammation (33); *PI16* (peptidase inhibitor 16) which may be a biomarker of loss of immune tolerance through its expression in Treg cell subsets (34). The difference between the level of *CD163* mRNA (2.2-fold upregulated in AdipTa+AdipTd) and the expression of the protein measured by IHC is noteworthy. We have no other explanation to offer than the respective sensitivity of the two methods. Of note also is the absence in the gene expression signature of the other monocytic-macrophage lineage marker *CD68* for which no difference by IHC between AdipN and AdipT was observed. The integrin signaling pathway includes an up-regulated *ITGB2* (β2-lntegrin) which mediates leukocyte recruitment into inflamed tissues (35); a down-regulated *ITGA6* (α6Integrin); *COL6A6* (collagen type VI alpha 6 chain) and *LAMA1* (laminin subunit alpha 1). The WNT signaling pathway includes: *PCDH12* (protocadherin 12); *TBL1X* (transducin beta like 1 X-linked) and *MYCL* proto-oncogene.

As the heterogeneity of clinical characteristics could have an impact on the gene expression profiles, we performed stratified analyses according to (1) receptor status: ER-positive (*n* = 27) or ER-negative (*n* = 8) tumor-bearing breasts and (2) menopausal status: menopaused patients (*n* = 27) (data not shown) to compare the sub-groups with AdipN samples (*n* = 5). When comparing the gene expression profiles of AdipN samples with adipose tissue (AdipTa+d) from ER-positive and ER-negative tumors, respectively, we observed that the list of upregulated genes differed by four genes. *PI16, CILP, VCAN*, and *LGMN* were up-regulated in adipose tissue from ER-negative samples, but not from ER-positive samples. *PI16, CILP, and LGMN* (described above) are among the genes involved in inflammation. *VCAN* encodes a chondroitin sulfate proteoglycan which is a major component of the extracellular matrix. The protein is involved in cell adhesion, proliferation, proliferation, migration and angiogenesis. With regard to the menopause status, the signature of differentially expressed genes between AdipN and AdipT is marginally affected by the selection of menopaused patient. Comparison of the top of the lists of upregulated genes in the two sets (menopaused vs. total samples) shows 16/18 genes shared by the two lists. The menopaused list has one additional (*PRG4*) and one missing (*LGMN*) gene.

## Discussion

The study presented here identifies parameters that distinguish adipose tissues from tumor-bearing breasts from those of tumor-free breasts. We termed these tissues as constitutional, as opposed to cancer-associated-adipose tissues (CAAs) present at the invasive front of the tumor, which undergo massive phenotypic alterations under active cross talks with the tumor. “adjacent-to-the-tumor” AdipTa samples were taken at a distance of 0.5–1 cm from the tumor, purposely discarding adipose tissues engaged in direct cross talks with the tumor including the CAAs. For control tissues, as we wanted to avoid any influence of the tumor, the ideal source of tissue would therefore have been the tumor-free contralateral breast tissue. Because this was not ethically acceptable, we instead sampled “distant from the tumor” AdipTd tissues at a distance > 5 cm from the tumor. As with the AdipTa samples, we aimed to prevent contamination by normal epithelial tissue, which would have confounded the interpretation of the secretomes and gene expression data.

Neither the size nor the density distinguishes adipocytes adjacent (AdipTa) or distant (AdipTd) to the tumor site from those of tumor-free breasts (AdipN). In addition, none of these samples displays crown-like structures detectable by macrophage specific staining for CD68 or CD163. Assuming that the structure of the fat tissue is homogeneous within the breast stroma, these observations imply that the tumors did not develop within obese-type stroma. In addition, the absence of CAAs in AdipTa sampled at a distance of 0.5–1 cm from the tumor means that the adipocyte-tumor cross talk is confined to an area <0.5 cm from the tumor.

The distribution of the IHC scores for immune infiltration and vascularization markers in tumor-bearing samples AdipTa and AdipTd does not differ from each other. It is neither significantly different from tumor-free samples AdipN, leading to the conclusion that the presence of the tumor at a distance of 0.5–1 cm does not affect the overall immune cell infiltration and vascularization of the stroma.

Neither secretion of adipocyte specific cytokines (leptin and adiponectin) nor angiogenesis factors (VEGF, HGF) or MCP1 and IL6 discriminate fat tissues from tumor-bearing (AdipTa or AdipTd) and tumor-free breasts (AdipN) (Table 4). By contrast, IL-8 secretion is associated with the presence of cancer (*p* = 0.01). When the AdipTa+AdipTd cohort is restricted to the 25 patients carrying ER+PR+ tumors instead of the 43 patients in the complete set, this result remains significant although with a diminished *p*-value (*p* = 0.03 in multivariate logistic regression analysis). The effect is likely due to the major reduction in sample size.

A search for association between IHC markers and secretome revealed that the mast cell tryptase (MCT) score correlated with IL-8 secretion capacity, suggesting that the secreted IL-8 may originate mostly from the mast cells compartment of the stroma. Co-IHC for MCT and IL-8 would be decisive to test this assumption. Recent data from Al-Khalaf et al. highlighted the key role of IL-8 in activating breast CAA and promoting their paracrine pro-tumorigenic effects (36). They show that breast cancer-associated adipocytes (CAAs), express higher level of IL-8 than tumor-counterpart adipocytes (TCAs). This result may appear to contradict our observations. However, it noteworthy that (i) their TCAs include both our AdipTa and AdipTd normal tissues, (ii) the level of IL-8 in TCAs although lower than in CAAs is not null, (iii) there is no comparison with adipocytes from healthy breast. The role of IL-8 in various types of cancers is well-documented (37–39). Within the tumor mass, IL-8 is produced by both cancer cells and components of the stroma such as adipocytes and mast cells. In breast cancers, high serum levels of IL-8 correlate with advanced clinical status (40). In a complex array of autocrine and paracrine functions including feedback loops, IL-8 signaling is involved in all the stages of tumor development including proliferation, angiogenesis, and inflammatory setup… Most importantly, it induces EMT in human carcinoma cells, associated with metastasis and stemness, thus increasing invasiveness and greater resistance to various cytotoxicities including immune destruction (39).

The comparison of gene expression in adipose tissue adjacent to or distant from the tumor showed no significant differences. By contrast, expression profiles in adipose tissue of tumor-free breasts clearly differed from that of cancer-bearing breasts. The differential signature includes a significant fraction of genes involved in inflammation mediated by cytokine, integrin and wnt signaling pathways. Besides these genes, which are assigned to established pathways, the list includes genes whose relevance to adipose tissue or cancer is not obvious. For example the *TPR* gene (translocated promoter region) which is at the top of the upregulated genes (x5.5). It is a nucleoporin component of the nuclear pore complex (NPC), involved in trafficking across the nuclear envelope. It was originally described as a partner in oncogenic fusions with the Met, TRK and RAF tyrosine kinase receptors in various cancers. It carries a wide range of nuclear functions, including nuclear transport, chromatin organization, regulation of transcription and mitosis (41). TPR is a spatial and temporal regulator of the spindle checkpoints. Its overexpression enhances the formation of multinucleated cells (42). *FBLN1* (Fibulin1) plays a role in cell adhesion and migration, and has been classified as potential tumor suppressor gene because of its capacity to suppress fibronectin-mediated inhibitory effects on cell attachment and spreading (43). *PODN* (Podocan) is highly homologous to members of the small leucine-rich repeat protein (SLRP) family, which are proteoglycans that regulate and maintain extracellular matrix collagen fibrils. It is highly expressed in adipocytes (44).

With regard to the heterogeneity of mammary tumors, we have performed an unsupervised analysis of gene expression within the AdipT cohort, which indeed shows heterogeneity but with poor gene signature that could not be assigned to any significant biological pathway (data not shown). Aware that the heterogeneity of the AdipT cohort could have an impact on the results of gene expression, we carried out the analysis on more homogeneous groups. Selecting the ER-positive samples has not a profound effect on the signature of differentially expressed genes. However, four genes from the signature obtained with the complete set are absent in the signature obtained with the ER-positive only samples. Interestingly, the four genes missing (*PI16, CILP, VCAN*, and *LGMN*) are at the top of the signature obtained with the ER-negative only samples. These four genes (*PI16, CILP*, and *LGMN*) represent only a fraction (3/8) of those involved in inflammation pathway in the Panther classification. Their expression pattern appears as a specific signature for adipose tissue in ER-positive tumor-bearing breasts. This correlation between the ER status of the tumor and the characteristics of the adipose environment leads to the assumption that permissiveness for a specific type of tumor can be determined by a specific stroma status.

Sturtz et al. (45) have reported results of a study with a similar design as ours in which they compared gene expression in fat tissues from tumor-free breasts or tumor-bearing breasts (adjacent or distant to the tumor). In this study, the distant samples were taken at >4 cm from the tumor, very much alike our study. Although the sites of adjacent samplings are not defined, the published H&E images of the distant and adjacent samples look very similar suggesting that the adjacent samples were quite distant from the tumors, certainly not close to the front. The authors report two differential signatures between samples from tumor-free and either tumor distant or adjacent tissues. The list of 20 differentially expressed genes at the top of Sturtz et al.' signatures shares 4 identical genes (*LGMN, C1QB, CD14, and CD163*) + 4 members of same gene family (*FBLN1, FBLN5, C1QC, and MS4A*) from our list of differentially expressed genes. The four common genes are part of the signature of inflammation. However, by contrast with our data, they also report a differential signature between samples distant and adjacent to tumor samples. Examination of the signatures displayed by distant or adjacent samples shows that they share 14/20 top genes, suggesting that distant and adjacent samples are indeed quite similar.

Overall, the data presented here show that constitutional fat tissue from tumor bearing breast display pro-inflammatory status parameters that distinguish it from healthy breast tissue. We recognize the exploratory nature of this study due to the limited size of the control cohort, the differences in subjects' median age and menopausal status between the two cohorts and the heterogeneity of tumor-bearing breast cohort. However, this should not hinder our conclusions drawn from statistically significant data. The report by Iyengar et al. (29) that menopause is a critical factor in adipose inflammation of the breast raised concerns about the significance of our findings. Indeed, 74.4% (32/43) women in our breast cancer patient group were menopausal as opposed to none in the non-cancer group. These authors associated inflammation status with two determinants: size of adipocytes and presence of crown-like structures (CLS). As discussed above, in our study neither the size nor the density distinguishes adipocytes from tumor-bearing breasts from those of tumor-free breasts. In addition, macrophage-specific staining for CD68 or CD163 did not reveal significant numbers of CLS in either group. The origin of these discrepancies may lie in differences in menopause status between their cohort and ours such as age at menopause, HST duration, cause of menopause (natural or oophorectomy), *BRCA1/2* status. Indeed, *BRCA* mutation carrier women were excluded from our cohort while they represent 39% of theirs.

The unique characteristics of constitutional fat tissues from tumor-bearing breast support the concept of cancer-permissive field. The question of whether these differences are constitutional or tumor-induced remains open, the two models not being necessarily mutually exclusive. However, since the crosstalk between the adipocytes and the tumor is limited to the immediate proximity of the tumor (10), the distant tissues are likely to be less influenced than the adjacent tissues by the effects of crosstalk. We have not observed differences between the two tissues.

It is noteworthy that the gene expression differential signature between AdipTa+AdipTd and AdipN does not include IL-8. This may appear paradoxical given that the tumor-bearing samples over-secrete IL-8 upon incubation *in vitro*. Perhaps the paradox is only apparent, given the different settings of measurements. On the one hand, gene expressions have been measured on snap frozen tissues right after surgical removal and on the other hand, secretome has been measured after 24 h incubation in culture medium *in vitro*. Although the steady state levels of IL-8 mRNA in AdipTa+AdipTd and AdipN are similar in tissues *in situ*, the differential potentiality for cytokines expression and secretion may be revealed by exposure to stressful culture conditions. Several reports support the hypothesis that expression of IL-8 may be stimulated by environmental stresses such as exposures to other cytokines, hypoxia, reactive oxygen species (ROS), and bacteria (46, 47). Furthermore, Bendrik and Dabrosin (48) have reported an increased secretion of IL-8 upon estradiol E2 incubation of normal human breast tissue biopsies *in vitro* and shown that mainly the epithelial cells were expressing IL-8. They also observed a significant correlation of E2 and *in vivo* extracellular IL-8 in human breast cancer. Therefore, it is tempting to propose a model in which permissiveness of human breast to cancer cell growth may be conditionally induced by estrogen stimulation or other kind of stress. In women, monthly estrogen exposure may uncover the status of cancer-permissiveness of the mammary gland stroma *via* cumulative exposure to IL-8 and other cytokines.

The concept of stroma niche-driven oncogenesis has been documented in other organs. Many cases of association of germline mutations in bone marrow microenvironment with dysregulated hematopoiesis have been reported (9). As an example, Kode et al. have shown that an activating mutation of β-catenin in mouse osteoblasts induces a “mutagenic” environment in myeloid and lymphoid progenitors leading to development of acute myeloid leukemia (49). Along the same lines, Zambetti et al. demonstrated that ligands secreted from mesenchymal cells such as pro-inflammatory endogenous damage-associated molecular pattern (DAMP) molecules S100A8 and S100A9 contribute *via* their binding to the toll-like receptor 4 (TLR4) to the induction of DNA damages in hematopoietic stem and progenitor cell in the mouse (50).

We would like to propose the hypothesis that permissiveness of human breast to the development of cancer can be determined at least in part by the constitutive status of adipose tissue. Whether this property can be translated into a risk prediction marker will require further work. In support of such a model, Magi-Galluzzi et al. have reported that gene expression profiles in normal tissue from tumor-bearing prostate can predict prostate cancer outcome and therefore be translated into a marker for aggressive disease (51). However, a significant difference between this study and ours should be pointed out regarding the nature of the niche and the cell autonomy mechanism of cancer development. Whereas, we describe a non-cell autonomous contribution of the stroma, Magi-Galluzzi's study identifies the niche within the cancer-target epithelial tissue itself, thus implying a cell-autonomous mechanism.

While the definition of a signature will require a larger sample size, we believe that our data provide support to the concept of constitutional permissiveness (predisposition) of stromal tissue to oncogenic-induced tumor growth. The investigation of genetic variations that modify the herein investigated molecular markers may provide additional information that will help assess the risk of breast cancer.

## Data Availability Statement

The original contributions presented in the study are publicly available. This data can be found here: Array-Express repository (http://www.ebi.ac.uk/arrayexpress) (accession number: E-MTAB-8638).

## Ethics Statement

The studies involving human participants were reviewed and approved by Gustave Roussy Institutional review board. The patients/participants provided their written informed consent to participate in this study.

## Author Contributions

JF, SD, PO, DS, J-PA, and BL: conceptualization. JF and IM: methodology. HP, JA, and PD: formal analysis. IM, DS, FD, MB, EL, M-CM, PV, and MH: investigations. JF: writing—original draft. JF, SD, DS, and J-PA: writing—review and editing. SD and JF: funding acquisition and supervision. CM: resources. All authors contributed to the article and approved the submitted version.

## Conflict of Interest

The authors declare that the research was conducted in the absence of any commercial or financial relationships that could be construed as a potential conflict of interest.
