# Supplementary material for: Adipose Tissue Properties in Tumor-Bearing Breasts
Source: Front Oncol. 2020 Aug 21;10:1506. doi: 10.3389/fonc.2020.01506 (PMC7472783; doi:10.3389/fonc.2020.01506)
Supplement: Supplementary file 1 [file Data_Sheet_1.pdf]

Supplemental Table S1: Comparison of secretion values by AdipTa and AdipTd (ELISA measurements were log2-transformed and paired t-tested) showing the high similarity between the two samples in each individual.

	Pearson r statistic	Pearson r p-value	Paired t-test p-value
Leptin	0.80	1.10^{-10}	0.5
Adiponectin	0.71	2.10^{-7}	0.3
VEGF	0.41	7.10^{-3}	0.8
HGF	0.53	2.10^{-4}	0.8
MCP1	0.60	2.10^{-5}	0.8
IL-8	0.50	7.10^{-4}	0.2
IL6	0.51	5.10^{-4}	0.4

Supplemental Table S2: P-values of associations between IHC scores and secretome. The association test was performed using linear models as described in the methods section. For example, the MCT score well predicts IL8 secretion (on the log2 scale) (p = 0.006). This is the strongest association we found. It is further illustrated on Figure 5B.

Secretion levels of / IHC expression of	MCP1	IL-8	IL6
CD34	0.64	0.27	0.84
CD68	0.81	0.33	0.71
CD163	0.38	0.09	0.21
MCT	0.02	0.006	0.03
