# Supplementary figures and images for: Adipose Tissue Properties in Tumor-Bearing Breasts
Source: Front Oncol. 2020 Aug 21;10:1506. doi: 10.3389/fonc.2020.01506 (PMC7472783; doi:10.3389/fonc.2020.01506)

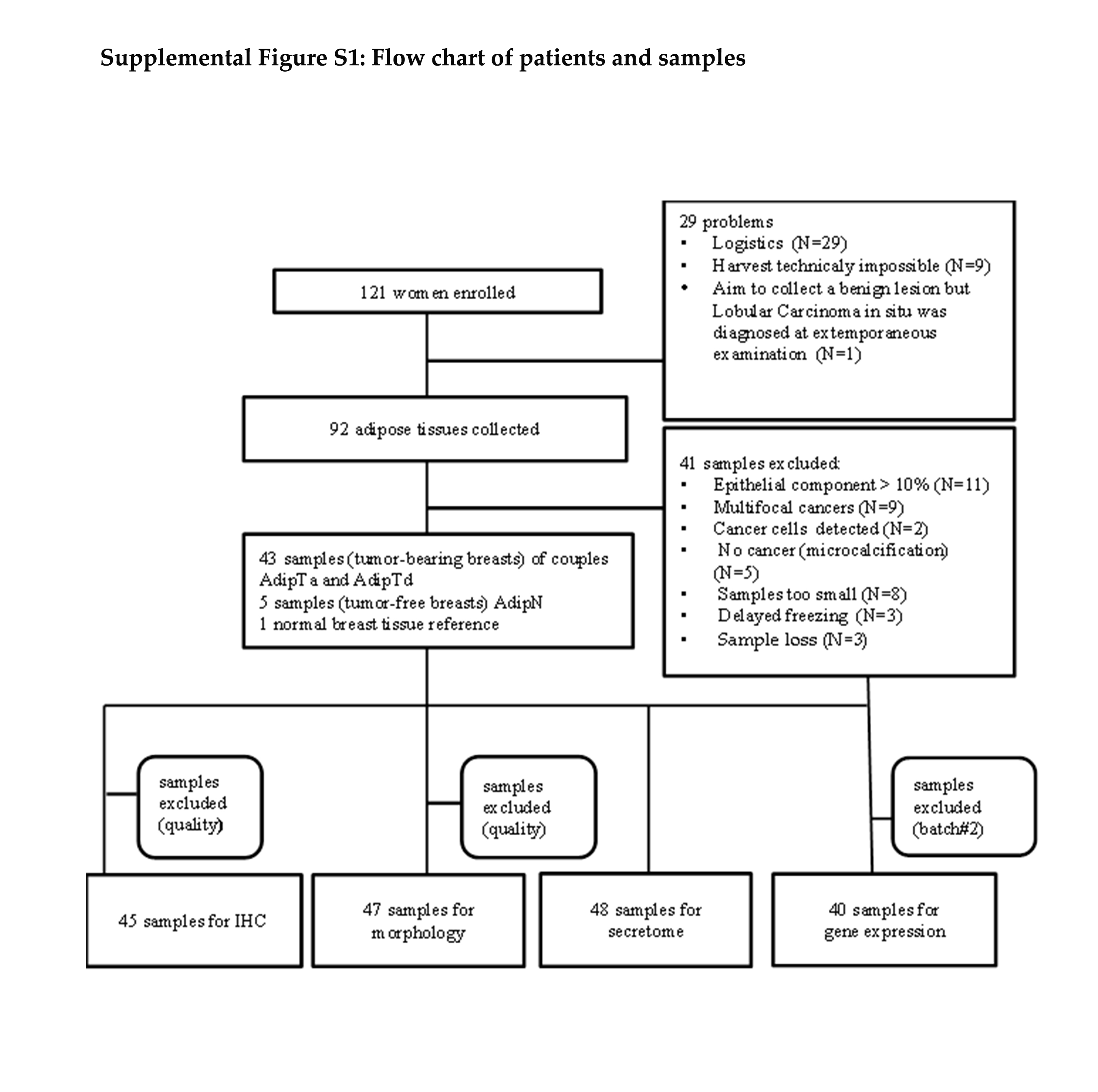

Supplement: Supplementary file 3 [file Image_1.TIF]

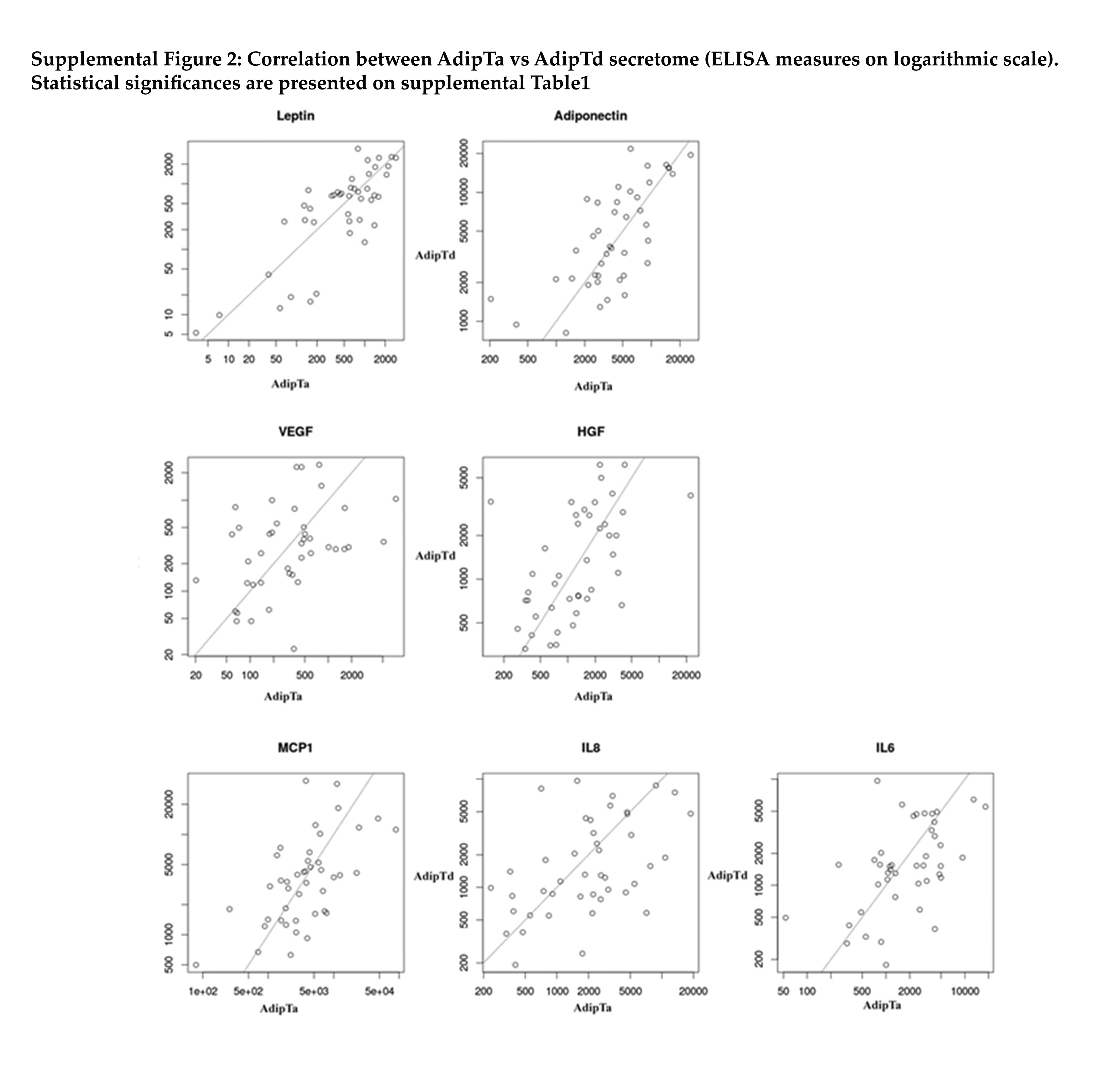

Supplement: Supplementary file 4 [file Image_2.TIF]
